# Book Review: Pragmatic Particles: Findings From Asian Languages

**DOI:** 10.3389/fpsyg.2021.736899

**Published:** 2021-09-30

**Authors:** Yi Shan

**Affiliations:** ^1^School of Foreign Studies, Nantong University, Nantong, China; ^2^College of Foreign Languages, Fujian Normal University, Fuzhou, China

**Keywords:** pragmatic particles, findings from Asian languages, Jieun Kiaer, London and New York, Bloomsbury Academic

Particles “play a crucial role in unfolding structural skeletons and making syntax predictable, yet at the same time enriching socio-pragmatic, interactional meanings. Particles are observed cross-linguistically as a complex of syntactic, semantic, and pragmatic primitives” (p.IX). Pragmatic particles, generally, facilitate structuring communication and embedding utterances into communicative context. It is not easy to give a precise definition of pragmatic particles, because these items serve various functions: (1) representing the speaker's attitude toward the content of the sentence (Kuno, 1973, p. 4), (2) modifying the illocutionary force, (3) indicating the beginning, continuation or end of a turn, (4) reflecting a coherence break in the ongoing discourse, or (5) marking the background or foreground status of a stretch of discourse. Although widely observed in the languages of the world, they are largely unexplored in theoretical linguistics and empirical investigations, because their general linguistic properties are often marginalized from an Anglo- or Euro-centric perspective. This book, *Pragmatic Particles: Findings from Asian Languages*, aims to fill this gap. The intended readers are scholars in the fields of pragmatics and morphosyntactic studies, as well as teachers and students engaged in the teaching and learning in these domains.

The book begins with the observation, description and explanation of particles like the interrogative particle *bro/waa* in Lao and the politeness particle *po*/ho in Tagalog logically, from a non-Euro-centric perspective. The observation of the common performances of particles of Asian languages, such as the honorific particle *-nim* in Korean and the honorific particle *app* in Hindi, reveals the role of socio-pragmatic motivations in shaping morphosyntactic variations [e.g., the plural marker *-(e)ra* in Bengali]. Accordingly, it is found that formal paradigms in contemporary Anglo-centric grammar fail to adequately scrutinize, depict and interpret the constructive roles (e.g., the disambiguation particle *-la* in Tibetan) and socio-pragmatically rich meanings of particles in these languages, and that the commonalities shared by these languages can contribute to linguistic theory.

The book comprises nine chapters. The highlights are the investigations of (1) the constructive role of particles in languages with flexible word orders and (2) various expressive and attitudinal meanings exhibited by particles which are easily subject to sociocultural factors, within the frameworks of Dynamic Syntax and expressive semantics (Potts, 2005). The aim is to reveal the complex nature of particles. Chapter 1 is an introduction in which Jieun Kiaer provides a brief overview of the status quo of research into Asian languages and particles, of the challenges that these languages can bring to contemporary linguistic theories, and of the target languages under discussion and their key characteristics. After this, the remaining eight chapters are organized in four parts, each reporting on issues related to certain aspects of particles. They include respectively the demonstration of how particles together with prosody and context build a syntactic structure (Chapters 2-3), the pragmatic realization of expressions and particles (Chapters 4-5), the hypothesis and grammatical framework adopted in the analysis of the particles in the Asian target languages (Chapter 6), and the analysis of the constructive, expressive and socio-pragmatic nature of particles (Chapters 7-9). The book wraps up with an epilog in which Kiaer summarizes and highlights the key issues dealt with in this work.

Chapter 1 provides a fairly lengthy introduction. In the brief overview of the status quo of research into Asian languages, Kiaer mentions Pānini's grammar as the formal foundation for contemporary linguistics, due to its influence on Saussure and Chomsky. However, as he claims, this Asian touch within linguistics has been lost, due to the focus on data mainly from European languages. This loss is attributed to the implicit assumption that “the linguistic consistencies found in these languages will be applicable to all other languages with little parametric variation” (p. 1). Thus, Kiaer points out the necessity of showcasing the frequently-overlooked properties of Asian languages, which are not exclusively exhibited in Asian languages, but are observed cross-linguistically among world languages. Opposing the view held by contemporary linguists that particles fulfill merely a single function-semantic or pragmatic, he argues that particles function as a grammatical entity which provides multiple dimensions of information, structural, semantic, and pragmatic. According to him, instead of being arbitrary and peripheral, particle behaviors are systematically and consistently motivated by socio-pragmatic needs. In other words, their behaviors are influenced by the speaker's desire to achieve efficiency, expressivity and empathy in social communication. Since Syntactic Structures (Chomsky, 1957), linguistic categories and attributes that are non-existent or less relevant in English and Western European languages have been less celebrated and explored. Particles are a good case in point. Actually, the whole set of meanings projected by them has not been systematically studied and properly underpinned in linguistic theories thus far. Focusing on Asian languages spoken in the Middle East, Central Asia, South Asia, Northeast Asia, and Southeast Asia, Kiaer aims to shed new light on the constructive and expressive roles played by particles in the syntax, semantics and pragmatics of natural language. He intends to show that the patterns of particles and argument realization in the target languages demonstrate that syntactic decisions are fundamentally driven by socio-pragmatic needs. Asian languages share some properties worth investigating. These features are systematic and pragmatic in nature, and (socio)-pragmatically driven, including flexible word orders, pragmatic realization of lexical expressions and particles, and rich socio-pragmatic and interpersonal meanings.

Part I begins with chapter 2 in which Kiaer tries to model flexible word orders in Asian languages, which on the whole show flexible structures, where grammatical relations are mostly realized through various sources including particles, rather than through word orders. In the following sections, he first discusses some of the key structural properties of the target languages, by showcasing mainly Korean along with some examples from other languages. The core structural properties of Korean include (1) flexible word order, (2) pragmatic realization of expressions, and (3) linear-order effect: left-to-right asymmetry. Word-order variation in Korean and many other Asian languages with the flexible ordering property known as “scrambling” (p. 31) challenges fundamental assumptions made in the Chomskian paradigm, because Korean and some other Asian languages allow very flexible NP ordering, and scrambling is not driven by the presence of any morphological trigger and is semantically vacuous (Saito, 1989). These traits run counter to the spirit of the current minimalist framework (Chomsky, 1995). Based on examples from Korean, Kiaer shows that nominal expressions can form a well-qualified constituent, even without postulating any verbal trace, which is applicable to other Asian languages. In these instances, the “surprising constituent” (Takano, 2002) can be moved together, or located away from its local interpretable domain, thus satisfying the movement criterion of constituency. The surprising constituent formation can be accounted for syntactically, by drawing on the assumption that the internal arguments (i.e., objects) are generated initially within the maximal projection of a main verb (VP), and the external argument (i.e., subject) is merged as Specifier (SPEC) of V (Koizumi, 2000), on the analysis that the external argument is raised to SPEC of intonational phrase (IP) or Tensed Phrase (TP) in overt syntax (Nemoto, 1993), and on the proposal of the adjunction-like oblique movement (Takano, 2002). Subsequently, Kiaer discusses how lexicalist frameworks such as HPSG (Head-driven phrase structure grammar), LFG (Lexical-functional grammar), and (multimodal) CCG (Combinatory categorial grammar) can deal with local and non-local word-order variation. Chapter 3 shows how natural language syntax is resource-sensitive, contrary to Chomsky's initial claims. In particular, it is shown that locality and incrementality matter in efficient structure building. Kiaer proposes that on the basis of maximizing the efficiency in communication, the ordinary speaker builds up the components of their sentences. From the angle of structure building, the essence of this efficiency is catalyzed as “locality”. In other words, people build structures locally, check locally, and then locally pack up their linguistic input–as they hear. Incrementality means that both structure-building and grammaticality-assessing and -assigning are achieved in an incremental manner within its local grammatical unit.

The two chapters in the second part of this book are concerned with the pragmatic realization of expressions and particles. Chapter 4 analyses a particle's roles in realizing multidimensional meanings, expressive, attitudinal and interpersonal. Potts' theory of expressive semantics is first introduced to show that particles are expressive, projecting multidimensional, expressive and attitudinal meanings. The linguistic competence of ordinary speakers in Asian languages is often measured by their ability to choose a set of particles appropriate to the socio-pragmatic context. Expressive semantics enables people to understand and formulate the rich array of meanings projected by particles in Asian languages. Based on Potts' expressive criteria, particles function as expressives. Kiaer compares the dynamic meanings of expressive particles in sentences to the “layers of an onion” (p. 82) with predicate content as the outer layer and expressive meaning providing the inner layers. Besides, the FTA (face-threatening act) theory and its limitations are discussed when it is used to explain Asian languages. Chapter 5 deals with the socio-pragmatic features of particles in Asian languages. As is pointed out by Kiaer, in Asia, seniority based on age and/or respect for one's elders or bosses within a community or workplace are crucial in building interpersonal relations. According to him, “honorifics” or “politeness” cannot adequately express the full range of possible interpersonal meanings. Thus, he introduces a lexical matrix to fully capture the interpersonal dynamics manifested in particles. He claims that particles utilized in the same utterance are not chosen at random, but rather they are employed to gradually build and reinforce socio-pragmatic meanings set between interlocutors. In line with this claim, particles are tuned and modulated throughout the utterance in both verbal and non-verbal channels of communication, to build interpersonal meanings. In order to explain this, he proposes the MMH (multimodal modulation hypothesis). Finally, he discusses the meanings and distributions of second-person pronouns and address terms in Asian target languages, which are far more complex than those found in Western European languages.

In Part III (Chapter 6), attention is shifted to particles as a meaning complex. Kiaer first shows some of the limits of particle research in Asian languages within contemporary linguistics, including (1) the limit of glossing particles, (2) -*un/nun* in Korean (K) and -*wa* in Japanese (J) as difficult-to-define topic markers, (3) limit of previous studies: prevalent uses of non-typical -*wa* (J) and -*un/nun* (K), (4) interchangeability of particles, (5) evidence from other Asian languages, (6) perspective particles, (7) attitudinal particles, and (8) subjective vs. objective particles. Then he discusses particle realization, which, for him, is a complex matter. In this discussion, he showcases examples of object particle realization from Korean and perspective particles from both Korean and Japanese to unravel the complexity of particle performance. In order to show that particles act as multidimensional meaning complexes rather than one-dimensional markers, Kiaer proposes a lexical matrix with four dimensions: constructive, mood and emotion, interpersonal, and style. Finally, he points out that although this chapter mainly focus on Korean examples, the method of adopting such lexical matrices can easily be applicable to other Asian languages.

Chapter 7, the first of Part IV, investigates pragmatic syntax. In this chapter, the DS (Dynamic Syntax; Kempson et al., 2001; Cann et al., 2005) formalism is introduced, to model the role of particles in syntactic building and the composition of socio-pragmatic meaning in the target languages. The core notion of DS is early under-specification of structure and its subsequent update at a later stage. In DS, sensitivity to linear order forms an integral aspect of structure-building. This linearity replaces a rigorously top-down hierarchy of configuration. Therefore, a structure is built incrementally along the left-to-right linear order. Based on this observation, Kiaer proposes the 3E (efficiency, expressivity and empathy) model: “ordinary language users constantly negotiate between these three desires in order to produce the most optimal linguistic behaviors relative to the given communicative environment” (p. 161). So, it is argued that the desire to make communication socio-pragmatically appropriate, empathetic, efficient, and expressive is crucial in comprehending any syntactic variation. Chapter 8 focuses on constructive particles and syntactic fluidity. In dealing with this topic, Kiaer first discusses the roles of constructive particles in flexible syntax, by citing examples from both A- and B-group languages^1^. After that, he illustrates how the LINK operation in DS can be used in explaining building an extended clause. There are numerous other structures allowing for flexible configuration of diverse, multiple pieces of information in an extended structure. The repeated employments of particles ensure that given expressions are to be construed in the same utterance domain rather than in different, independent domains. This rule of locality enables people to piece together fragmentary information in the same local structure to build and enrich the existing meanings rather than to initiate independent structures. As a result, syntactic structure is built across users in a seamless and predictable manner. This, in Kiaer's point of view, reflects human innate syntactic competence, which should be modeled as part of linguistic theory. Chapter 9 explores diverse expressive and attitudinal particles in Asian languages. By citing evidence from four case studies using Thai, Tagalog, Chinese and Japanese, Kiaer proposes the socio-pragmatic attributes of particles. The meanings expressed by utterance-initial, -medial, and -final particles in different Asian languages have to be modulated and remain consistent throughout an utterance. Finally, he utilizes the LINK operation in DS to show how expressive and attitudinal meanings can be integrated in ongoing proposition building and to model the multimodal, multidimensional meanings exhibited by particles.

Rounding off the nine chapters of the book, Kiaer presents the epilog in which he sums up and attaches importance to the key topics discussed in the previous chapters, including (1) the syntactic fluidity and semantic/pragmatic diversity that are particularly visible in socio-pragmatic meaning constructions, (2) the ignorance of fluid structures and oversimplification of multidimensional meanings due to Euro-centricism, (3) the conceptional flaw of contemporary theoretical linguistics–pursuing logical foundations in a vacuum, (4) the multimodal modulation of multidimensional meanings, (5) the proposal of using translanguaging as the conceptual foundation for linguistic modeling, etc.

As a complex of syntactic, semantic, and pragmatic primitives widely observed in the languages of the world, particles are largely unexplored in Anglo-centric grammar formalisms, which are inadequate to properly observe, describe and explain the structure-building roles and rich socio-pragmatic meanings projected by the particles in Asian languages. This book fills the gap between formal and functional, theoretical, and empirical approaches to language, by dealing with the formal and functional foundations of Asian languages from a holistic perspective. Using particles from Asian languages to challenge prevalent Euro-centric views on verb-centric, sentence-bound analyses, the author asserts that Asian languages ought to greatly impact the advancement of linguistic theories, and that socio-pragmatic attributes of these particles play a key role in explicating their grammatical performance.

With respect to organization, the overall structure of the book is highly effective with an insightful introduction and a penetrating epilog. The inclusion of introductory paragraphs in most of the chapters and a reader-friendly summary at the end of each chapter is especially useful, in terms of guiding the reader through each chapter. However, instead of covering the full range of dialogue data, this book just analyzes particles in single utterances. This is a minor shortcoming, because the modulation of multidimensional meanings, multimodal, emotional and attitudinal, across speakers depends on much more complex and dynamic context than on a single utterance.

All in all, this is an inspirational book which is informative and practical. Through an easily accessible approach to the subject, this book gives new insights to readers, who have a keen interest in the socio-pragmatic elements of language and linguistic theories beyond the Anglo-centric discussion. It significantly contributes to theoretical linguistics, pragmatics and morphosyntactic studies in particular, and the study of particles in Asian languages.

## Author Contributions

The author confirms being the sole contributor of this work and has approved it for publication.

## Funding

Humanities and Social Sciences Youth Foundation, Ministry of Education, China, Grant number: 16YJC740011.

## Conflict of Interest

The author declares that the research was conducted in the absence of any commercial or financial relationships that could be construed as a potential conflict of interest.

## Publisher's Note

All claims expressed in this article are solely those of the authors and do not necessarily represent those of their affiliated organizations, or those of the publisher, the editors and the reviewers. Any product that may be evaluated in this article, or claim that may be made by its manufacturer, is not guaranteed or endorsed by the publisher.

## Footnotes

^1^A group: Flexible word orders with rich case particles, pragmatic realization of expressions (i.e., pro-drop), in general particle-rich languages such as Arabic, Korean, Japanese, Tamil, Hindi, Urdu, Bengali, Tibetan, Tagalog, Turkish, Persian, Mongolian, and Sanskrit (most of them except Standard Arabic show strong verb-finality). B group: Flexible word orders yet no case particles, pragmatic realization of expression (i.e., pro-drop), in general particle-rich languages, mostly SVO order preferred, as in Mandarin Chinese, Thai, Indonesian, Vietnamese, Khmer, Lao, and Burmese (SOV preferred).
